# Association of Hyperlipidemia and Hyperglycemia With Cognitive Function in Type 2 Diabetes: A Cross-Sectional Analysis

**DOI:** 10.7759/cureus.72227

**Published:** 2024-10-23

**Authors:** Tasneem Ansari, Manish Sawane

**Affiliations:** 1 Physiology, NKP Salve Institute of Medical Sciences and Research Centre and Lata Mangeshkar Hospital, Nagpur, IND

**Keywords:** cognition, glycated hemoglobin, hyperlipidemia, lipid profile, type 2 diabetes

## Abstract

Introduction

Cognitive impairment is increasingly recognized as a significant health concern, particularly in the early stages of diabetes. The type and severity of cognitive deficits may vary with the duration of diabetes and the effectiveness of glucose management. Complications associated with metabolic syndrome may exacerbate these cognitive declines. This study investigates the association of hyperlipidemia and hyperglycemia with cognitive function in patients with type 2 diabetes (T2D).

Methods

We conducted a cross-sectional study on type 2 diabetic patients aged between 30 and 40 years of age and having the disease for less than 10 years duration. We collected anthropometric measurements, tested glycated hemoglobin (HbA1c) and fasting lipid profiles, and assessed cognitive function using the Montreal Cognitive Assessment (MoCA).

Results

All participants exhibited elevated HbA1c levels and abnormal lipid profiles. We observed weak positive correlations between the duration of diabetes and low-density lipoprotein cholesterol (LDL; 0.448), very low-density lipoprotein cholesterol (VLDL; 0.398), total cholesterol (0.526), and HbA1c (0.360). There were moderately negative correlations between the duration of diabetes and MoCA scores (-0.522) and weak negative correlations between LDL and MoCA (-0.304), VLDL and MoCA (-0.259), and total cholesterol and MoCA (-0.409). The correlation between HbA1c and MoCA was also moderately negative (-0.779). Regression analysis revealed statistically significant associations of MoCA with the duration of diabetes, HbA1c, and lipid parameters, with HbA1c being the largest contributor to cognitive decline at 60.66%, while the contributions of various lipid parameters were considerably lower (LDL: R² = 0.092, VLDL: R² = 0.067, total cholesterol: R² = 0.167). The contribution of the duration of diabetes (R² = 0.272) to cognitive decline was less than that of HbA1c but more than the lipid parameters.

Conclusions

The findings suggest that hyperglycemia and the duration of diabetes are the major factors contributing to cognitive decline in patients with T2D. Patients should be advised to maintain optimal glycemic control and engage in activities that enhance cognitive function to prevent cognitive impairment. Regular cognitive screening for diabetic patients is also recommended.

## Introduction

Millions of people worldwide suffer from cognitive impairment, a primary factor affecting mental health. Diabetes is a notable risk factor for dementia in both animal models and humans. Specifically, type 2 diabetes (T2D) is associated with a 50% increased risk of dementia compared to type 1 diabetes, affecting attention, motor skills, executive functions, verbal memory, and processing speeds [1,2]. Hyperglycemia, a component of metabolic syndrome, shows the strongest link to the risk of developing cognitive impairment [3]. Commonly observed in individuals with T2D and prediabetes, diabetic dyslipidemia (lipid abnormalities) may play a significant role in the progression of Alzheimer’s disease, although findings are mixed [4-10]. For example, studies have shown that both low and high total cholesterol levels could influence the risk of Alzheimer’s disease and cognitive impairment, respectively [6,7]. Similarly, the relationship between Alzheimer’s disease and levels of triglycerides, high-density lipoprotein cholesterol (HDL-C), and low-density lipoprotein cholesterol (LDL-C) has yielded inconsistent results [8-10]. Despite these conflicting outcomes, the potential link between dyslipidemia and cognitive impairment cannot be dismissed.

Diabetes, which involves both hyperglycemia and dyslipidemia, can lead to cognitive impairment, but the relative contribution of each factor remains unclear. With the increasing prevalence of T2D and longer life expectancies, cognitive impairment related to diabetes could significantly impact future health resources. This study aims to determine the association of hyperlipidemia and hyperglycemia with cognitive function in T2D.

## Materials and methods

This observational, descriptive, cross-sectional study was conducted at the NKP Salve Institute of Medical Sciences and Research Centre and Lata Mangeshkar Hospital, Nagpur, India, and received approval from the Institutional Ethics Committee, NKP Salve Institute of Medical Sciences and Research Centre and Lata Mangeshkar Hospital (approval no. 06/2019). We enrolled 82 patients diagnosed with T2D, aged between 30 and 40 years, who had the disease for less than 10 years of duration. All participants provided written informed consent after receiving detailed information about the study. We collected a detailed medical history, recorded the anthropometric parameters, and calculated body mass index (BMI). Exclusion criteria included pregnancy, BMI over 30 kg/m^2^, other comorbid conditions (stroke, hypertension, ischemic heart disease, hypothyroidism, and mental illness), use of lipid-altering medications, and history of alcohol, tobacco consumption, or smoking. Repeated questioning and a detailed history were taken to ensure that the inclusion and exclusion criteria were rigorously applied to all the participants [11]. As a result, out of the 82 participants who were enrolled in the study, five of them gave a recent history of weight gain while three complained of recent weight loss, and three of the participants on repeated questioning admitted that they occasionally consumed alcohol. So, these 11 subjects were excluded from the study. Finally, the study was carried out on 71 diabetic subjects.

Participants were instructed to fast overnight before blood samples were collected under aseptic conditions and quality control measures. We collected 5 ml of venous blood and sent it for biochemical analysis. Glycated hemoglobin (HbA1c) levels were determined using an enzymatic method (reference range: 4% to 6%), and lipid profiles were measured using a Siemens' Dimension EXL automated machine (Siemens Healthineers, Germany) (biological reference intervals: total cholesterol of 200 mg/dl; triglycerides of 150 mg/dl; HDL of >40 mg/dl; LDL of <130 mg/dl; and very low-density lipoprotein cholesterol (VLDL) of 5-40 mg/dl).

To assess cognitive function, we used the Montreal Cognitive Assessment (MoCA), which scores up to 30 points across seven cognitive domains: language (3 points), abstraction (2 points), orientation (6 points), visuospatial/executive (5 points), naming (3 points), memory (5 points for delayed recall), and attention (6 points). An additional point is awarded for subjects with ≤12 years of education. A score above 26 is considered normal. We recorded the data in a Microsoft Excel spreadsheet and used IBM SPSS Statistics for Windows, Version 29 (Released 2021; IBM Corp., Armonk, New York, United States), for statistical analysis of the results.

## Results

The study was carried out on 71 T2D patients, of whom 58 were male and 11 were female. In all patients with T2D, HbA1c levels exceeded the reference range (normal: 4-6 gm%). The average glycated hemoglobin levels obtained were 8.08 mg%, suggesting that all of them had poor glycemic control. Similarly, lipid profile values were elevated, including LDL-C, VLDL-C, and total cholesterol. Correlation was studied between the various parameters as shown in Table 1. A p-value less than 0.05 was considered significant.

**Table 1:** Correlation between various parameters Abbreviations and normal reference range for various parameters: T2D: type 2 diabetes; LDL: low-density lipoprotein (normal: <130 mg%); VLDL: very low-density lipoprotein (normal: 5-40 mg%); total cholesterol (desirable: <200 mg%); HDL: high-density lipoprotein (40-60 mg%); MoCA: Montreal Cognitive Assessment; HbA1c: glycated hemoglobin (normal: 4-6 gm%); NA: not applicable

Parameters p-value cross tab analysis	T2D duration	LDL	VLDL	Total cholesterol	HDL	MoCA	HbA1c %
T2D duration	1	NA	NA	NA	NA	NA	NA
LDL	0.4485	1	NA	NA	NA	NA	NA
VLDL	0.3984	0.6601	1	NA	NA	NA	NA
Total cholesterol	0.5263	0.6752	0.7383	1	NA	NA	NA
HDL	-0.0226	0.0104	-0.0435	-0.1574	1	NA	NA
MoCA	-0.5221	-0.3042	-0.2594	-0.4087	0.0042	1	NA
HbA1c %	0.3603	0.1103	0.0486	0.1139	-0.0119	-0.7788	1

We observed weak positive correlations between the duration of diabetes and LDL-C (0.448), VLDL-C (0.398), total cholesterol (0.526), and HbA1c (0.360). Conversely, a moderately negative correlation existed between the duration of diabetes and the MoCA scores (-0.522). Weak negative correlations were also noted between LDL-C and MoCA (-0.304), VLDL-C and MoCA (-0.259), and total cholesterol and MoCA (-0.409). The correlation between HbA1c and MoCA was moderately negative (-0.779). These findings suggest that as the duration of diabetes increases, so do HbA1c, LDL-C, VLDL-C, and total cholesterol levels, while cognitive function declines. Furthermore, increases in LDL-C, VLDL-C, and total cholesterol are associated with decreases in cognition, as are increases in HbA1c levels.

To determine which factors contribute most to cognitive decline, we applied linear regression analysis with a 95% confidence interval between MoCA as a dependent variable and various other parameters as independent variables. The values of R^2^ and significance F are displayed in Table 2.

**Table 2:** Regression analysis between MoCA and various other parameters T2D: type 2 diabetes; LDL: low-density lipoprotein; VLDL: very low-density lipoprotein; HDL: high-density lipoprotein; MoCA: Montreal Cognitive Assessment; HbA1c: glycated hemoglobin

	R square	Significance F
MoCA and HbA1c	0.606676122	2.04261E-15
MoCA and LDL	0.092552415	0.010452302
MoCA and VLDL	0.067331883	0.030066659
MoCA and total cholesterol	0.167058762	0.000443307
MoCA and HDL	1.79194E-05	0.972255684
MoCA and duration of diabetes	0.272623142	2.32536E-05

The results indicate that the association of MOCA with various lipid parameters like LDL (significance F = 0.001), VLDL (significance F = 0.03), and cholesterol (significance F = 0.0004) was statistically significant but their contribution to cognitive decline was very lesser percentage (LDL (R^2 ^= 0.092), VLDL (R^2 ^= 0.067), and cholesterol (R^2 ^= 0.167)). Of the three, cholesterol (16%) contributed more. A statistically significant association was also found between MoCA and HbA1c (significance F: 2.04261E-15), and the value of R^2^ suggested its highest contribution to cognitive decline at 60.66% (R^2 ^= 0.6066). Another finding of our study was a statistically significant association between MOCA and the duration of diabetes (significance F = 2.32536E-05). However, its contribution to cognitive decline was 27% (R^2^ = 0.272), which was less than glycated hemoglobin but more than the lipid profile parameters.

**Figure 1:**
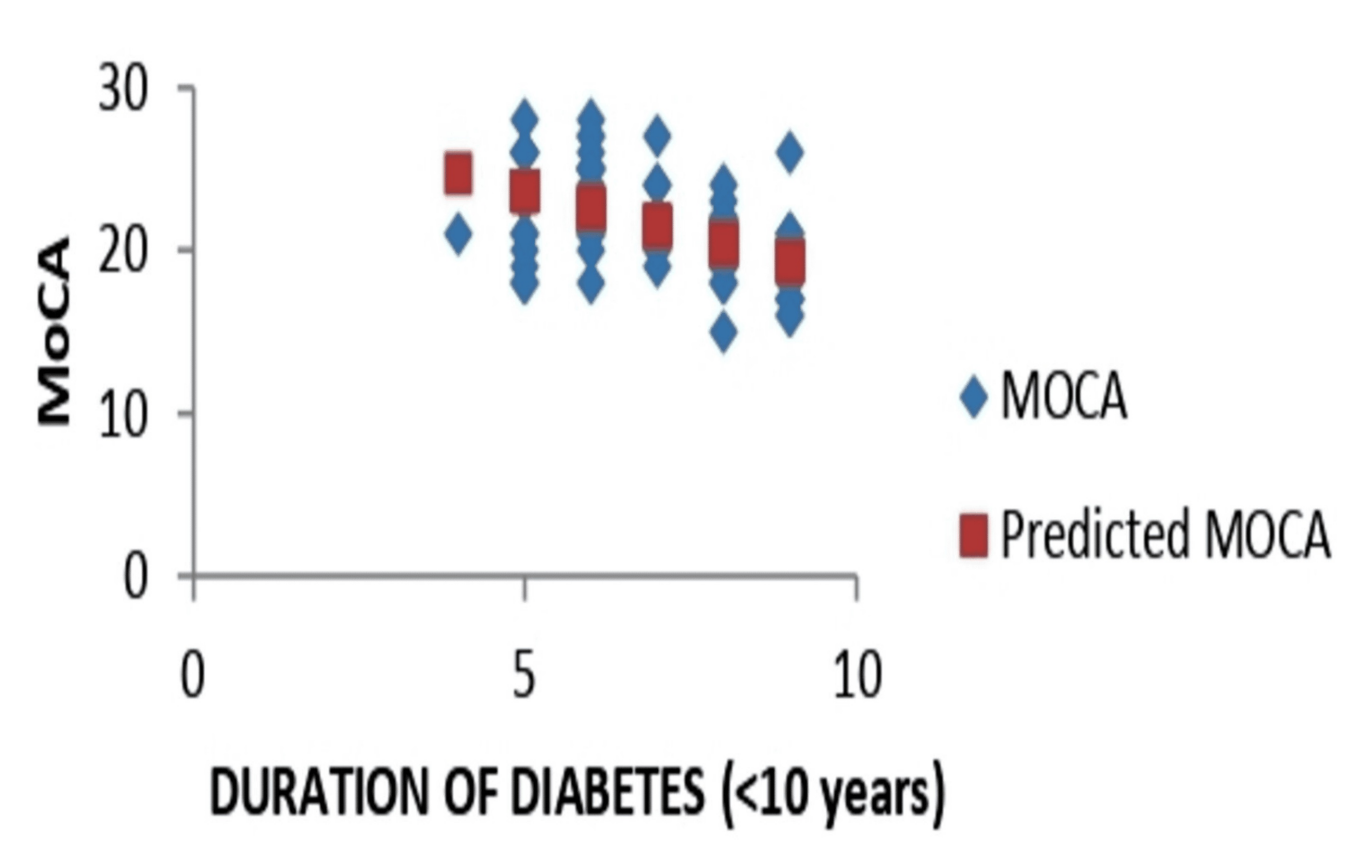
Duration of diabetes line fit plot MoCA: Montreal Cognitive Assessment

The scatter plot depicting the line of best fit and showing the relationship between MoCA and duration of diabetes is shown in Figure 1.

**Figure 2:**
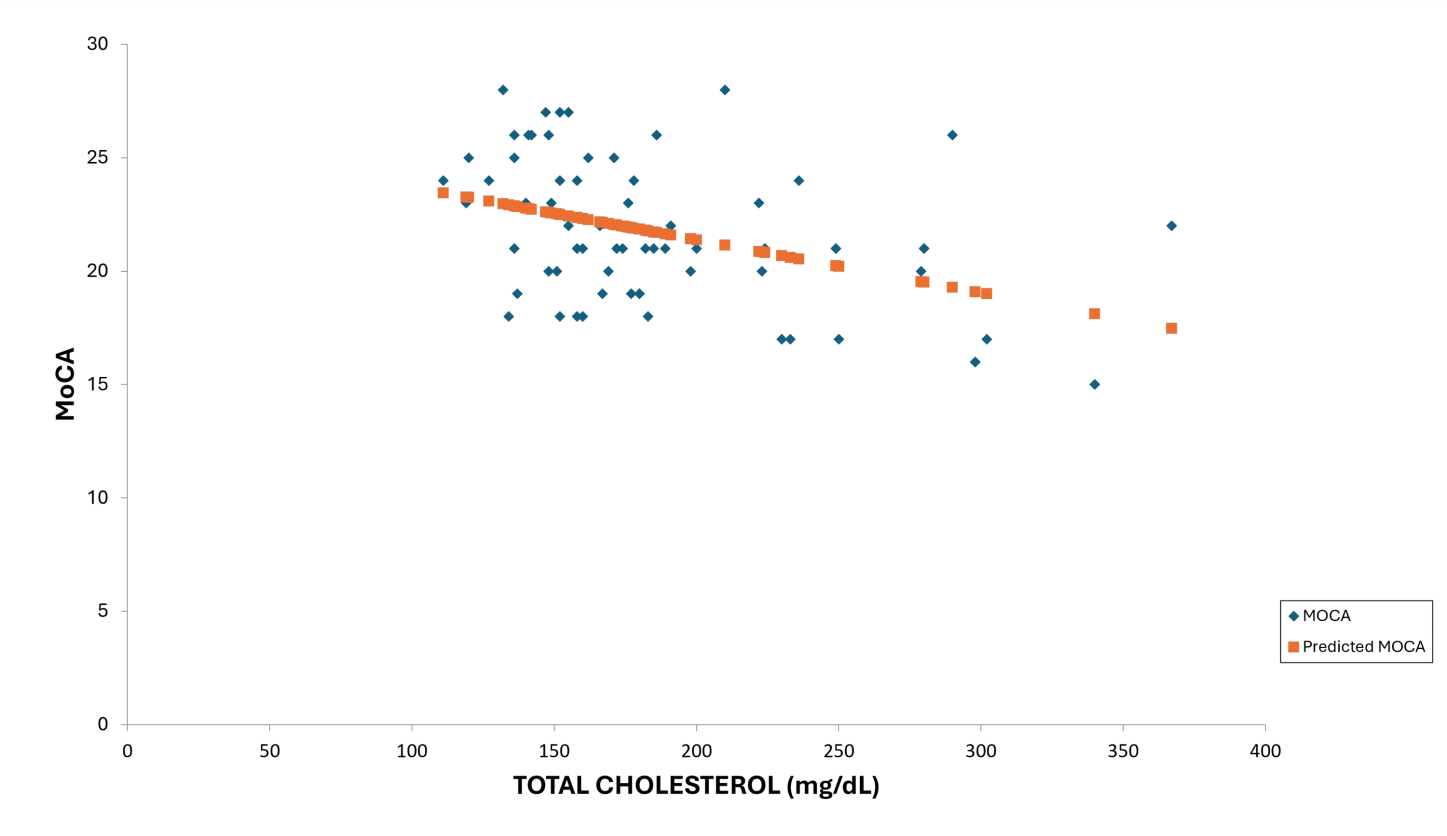
Total cholesterol line fit plot MoCA: Montreal Cognitive Assessment

As can be seen, a negative correlation was observed. Figure 2 shows the line of best fit for MoCA and total cholesterol.

**Figure 3:**
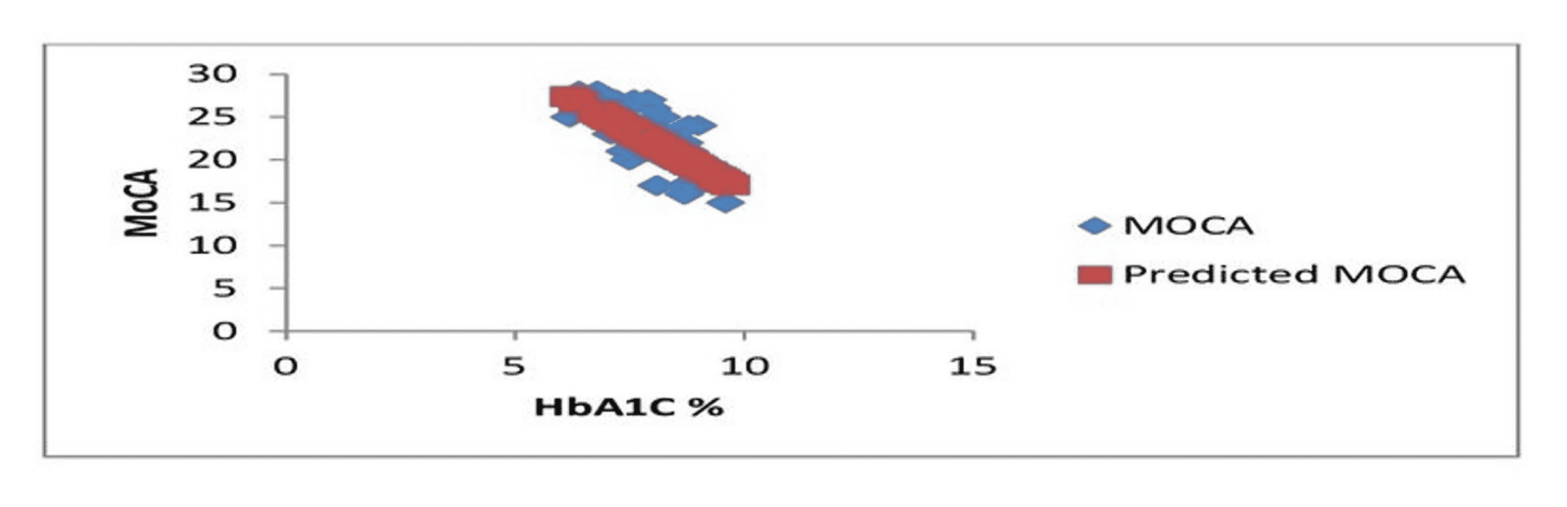
HbA1c % line fit plot MoCA: Montreal Cognitive Assessment; HbA1c: glycated hemoglobin

Again, the slope suggests a negative correlation. Figure 3 exhibits the line of best fit for MoCA and HbA1c.

The slope shows a negative correlation between the two.

## Discussion

In all patients with T2D, HbA1c levels exceeded the reference range (normal: 4-6 gm%). The average glycated hemoglobin levels obtained were 8.08 mg%, suggesting that all of them had poor glycemic control. Many of the participants visiting the tertiary care hospital where the study was conducted came from rural areas. They were ignorant about how to maintain their blood sugar levels. Even during the selection process, while taking detailed histories, many of the participants admitted to irregularities in consumption and non-compliance with drugs. This could be the reason for the rise in glycated hemoglobin levels. Poor glycemic control is consistent with a hyperglycemic state, which may explain the positive correlations between the duration of diabetes and both HbA1c levels and lipid parameters. Similar patterns have been documented in previous studies [12,13]. It has been explained in various literature that insulin plays a key role in triglyceride, LDL, and free fatty acid metabolism and there is enough evidence suggesting that diabetic dyslipidemia correlates strongly with hyperglycemia and hyperinsulinemia due to insulin resistance in T2D patients [14,15]. Notably, the duration of diabetes also correlated positively with cognitive decline, indicating that a longer duration of the disease may increase the risk of cognitive impairment [16,17].

To study which parameter contributes more to cognitive decline, linear regression analysis between MoCA as a dependent variable and other parameters as independent variables was studied. The values show that HbA1c (significance F = 2.04261E-15; R^2^ = 0.6066) had the most substantial impact, contributing 60.66% to cognitive decline. A statistically significant association between MOCA and duration of diabetes (significance F = 2.32536E-05) was also seen. However, its contribution to cognitive decline was 27%, which was less than glycated hemoglobin but more than the lipid profile parameters (R^2^ = 0.272). The associations between MoCA and lipid parameters (LDL-C (R² = 0.092; significance F = 0.01045), VLDL-C (significance F-0.03006; R^2^ = 0.067), and total cholesterol (significance F = 0.00044; R^2^ = 0.167)) were also significant, albeit contributing less to cognitive decline. The values show that of all the lipid parameters, cholesterol contributed more to cognitive decline. The scatter plot depicting the line of best fit and exhibiting the relationship between MoCA as a dependent variable and duration of diabetes, HbA1c%, and total cholesterol as independent variables shows a negative correlation with all three. Thus, it is clear that as the duration of the disease increases, cognition declines. Similarly, dyslipidemia and impaired glycemic control will also affect cognition in a negative way. However, the values obtained suggest that raised glycated hemoglobin affects cognition more substantially, followed by duration of diabetes. Prior research has produced mixed results regarding the relationship between lipid levels and cognitive function, with some studies indicating that high total cholesterol may increase the risk of Alzheimer’s disease in the elderly [18], while others suggest this association might only apply to middle-aged or male individuals [19,20]. Our findings indicate that HbA1c and the duration of diabetes are more closely associated with cognitive impairment in T2D patients than lipid parameters, contributing more significantly to cognitive decline. Despite numerous hypotheses and potential pathologic mechanisms proposed for cognitive impairment in diabetes - ranging from vascular disease, hypoglycemic events, direct insulin effects on the brain, oxidative stress, to advanced glycation end products - the exact pathways remain unclear.

This study had several important limitations. Firstly, its cross-sectional design restricts our ability to infer causality between diabetes management factors and cognitive impairment. Longitudinal studies are needed to establish temporal relationships and causation. Secondly, the sample was restricted to individuals aged 30 to 40 years, which may limit the generalizability of the findings to older adults who typically exhibit a higher risk for both cognitive decline and T2D complications. Additionally, the study did not account for variations in diet, physical activity, and other lifestyle factors that could significantly affect both glycemic control and cognitive health. The absence of data on these variables prevents a comprehensive understanding of the interplay between lifestyle factors and the biomarkers studied. Furthermore, the study did not consider genetic predispositions that could influence both the progression of T2D and the risk of developing cognitive impairments. Lastly, subtle cognitive changes might have been overlooked due to the reliance on biochemical markers and MoCA scores without incorporating more diverse and sensitive neuropsychological tests, limiting the depth of cognitive assessment.

## Conclusions

This study shows that hyperglycemia and the duration of diabetes are closely associated with cognitive impairment in patients with T2D. These patients should be informed of this risk and encouraged to improve cognition, such as by maintaining HbA1c levels within target ranges and undergoing regular cognitive assessments. Extensive longitudinal studies are needed to validate the pathogenic pathways and further elucidate the mechanisms leading to cognitive impairment in these individuals.
